# Climatic and socio-economic factors supporting the co-circulation of dengue, Zika and chikungunya in three different ecosystems in Colombia

**DOI:** 10.1371/journal.pntd.0009259

**Published:** 2021-03-11

**Authors:** Jasmine Morgan, Clare Strode, J. Enrique Salcedo-Sora

**Affiliations:** 1 Department of Biology, Edge Hill University, Lancashire, United Kingdom; 2 Institute of Systems, Molecular and Integrative Biology, University of Liverpool, Liverpool, United Kingdom; Louisiana State University, UNITED STATES

## Abstract

Dengue, Zika and chikungunya are diseases of global health significance caused by arboviruses and transmitted by the mosquito *Aedes aegypti*, which is of worldwide circulation. The arrival of the Zika and chikungunya viruses to South America increased the complexity of transmission and morbidity caused by these viruses co-circulating in the same vector mosquito species. Here we present an integrated analysis of the reported arbovirus cases between 2007 and 2017 and local climate and socio-economic profiles of three distinct Colombian municipalities (Bello, Cúcuta and Moniquirá). These locations were confirmed as three different ecosystems given their contrasted geographic, climatic and socio-economic profiles. Correlational analyses were conducted with both generalised linear models and generalised additive models for the geographical data. Average temperature, minimum temperature and wind speed were strongly correlated with disease incidence. The transmission of Zika during the 2016 epidemic appeared to decrease circulation of dengue in Cúcuta, an area of sustained high incidence of dengue. Socio-economic factors such as barriers to health and childhood services, inadequate sanitation and poor water supply suggested an unfavourable impact on the transmission of dengue, Zika and chikungunya in all three ecosystems. Socio-demographic influencers were also discussed including the influx of people to Cúcuta, fleeing political and economic instability from neighbouring Venezuela. *Aedes aegypti* is expanding its range and increasing the global threat of these diseases. It is therefore vital that we learn from the epidemiology of these arboviruses and translate it into an actionable local knowledge base. This is even more acute given the recent historical high of dengue cases in the Americas in 2019, preceding the COVID-19 pandemic, which is itself hampering mosquito control efforts.

## Author summary

Viruses transmitted by *Ae*. *aegypti* mosquitoes (dengue, Zika, chikungunya) are amongst the most significant public health concerns of recent years due to the increase in global cases and the rapid spread of the vectors. The primary method of controlling the spread of these arboviruses is through mosquito control. Understanding factors associated with risk of these viruses is key for informing control programmes and predicting when outbreaks may occur. Climate is an important driver in mosquito development and virus reproduction and hence the association of climate with disease risk. Socio-economic factors contribute to perpetuate disease risk. Areas of high poverty have abundance of suitable habitat for *Ae*. *aegypti* (e.g. due to poor housing and sanitation). This study investigated the factors affecting arbovirus incidence in three distinct regions of Colombia: Bello, Cúcuta and Moniquirá. The results show significant relationships between disease incidence and temperature, precipitation and wind speed. A decline in dengue following outbreaks of Zika (2016) is also reported. Measures of poverty, including critical overcrowding and no access to improved water source were also found to be higher in areas of higher disease incidence. The results of this study highlight the importance of using a multifactorial approach when designing vector control programs in order to effectively distribute local health care resources.

## Introduction

Vector-borne diseases are one of the most significant public health burdens globally, with 80% of the total world population at risk [1]. Arboviruses, including dengue, Zika and chikungunya, are of particular concern due to the recent increase in global cases promoted by the rapid spread of both their primary mosquito vector *Aedes aegypti* as well as their secondary vector *Aedes albopictus* [2]. Dengue infection can be asymptomatic but clinical presentations range from mild dengue fever (DF), a febrile illness similar to influenza, to the severe forms of dengue; dengue shock syndrome (DSS) and dengue haemorrhagic fever (DHF) [3]. Most Zika (ZIKV) infections are asymptomatic, with only approximately 20% of infections causing symptoms [4,5]. The clinical presentations of symptomatic ZIKV can include Zika fever, congenital Zika syndrome and Guillain-Barré syndrome. Congenital Zika syndrome refers to a group of birth defects, notably Microcephaly, which have been associated with ZIKV infection during pregnancy [6,7]. Infection with the chikungunya virus (CHIKV) is characterised by sudden onset fever, rash and arthralgia [8]. Joint pain associated with CHIKV is debilitating and whilst typically lasting a few days can last for many months or even years [9].

Dengue causes an estimated 390 million infections per year and has a distribution that covers every continent of the world with the exception of Antarctica [10]. The number of global dengue cases reported to the WHO has increased 15 fold over the last 20 years, with deaths also seeing a significant increase (4-fold) [11]. The first epidemics of ZIKV were reported in Yap, Micronesia (2007) and French Polynesia (2013); outbreaks were reported in Brazil in 2015 and 2016 which then led to a rapid spread of ZIKV to 48 countries within the Americas and the Caribbean [12]. ZIKV epidemics have also been reported in Singapore [13], Vietnam [14], Thailand [15] and Cape Verde [16]. CHIKV was first reported in Tanzania in 1952 and has since rapidly spread across the globe causing sporadic and significant epidemics in Asia, India, Europe and The Americas [17]. The epidemiology of CHIKV is notable due the sporadic patterns of outbreaks, likely caused by introduction of the virus into urban environments from the sylvatic cycle with nonhuman primates the most likely major reservoir host [18–20].

Spreading from its ancestral home in West Africa 400–500 years ago via the slave trade *Ae*. *aegypti* is found in tropical and sub-tropical regions [21]. Meteorological conditions directly influence the incidence of arboviruses by modulating vector mosquito populations. Conditions favourable to *Ae*. *aegypti* include an ideal temperature range between 20–35°C for mosquito development, fertilisation and vector competence [22]. Increases in temperature also increase viral replication rates within *Ae*. *aegypti*, increasing viral load and hence reducing extrinsic incubation periods and increasing transmission [23]. Non-climatic factors promoting *Ae*. *aegypti* populations include vegetation index, urbanisation and accessibility to human populations [24,25]. Latin America is significantly affected by *Ae*. *aegypti* borne viruses due to its habitat suitability for the vector, tropical climate and often limited medical resources and vector control programmes [26,27]. Colombia, located in the north-west corner of South America, has 140,612 km^2^ of suitable habitat for *Ae*. *aegypti* throughout the country based on the presence of climatic characteristics [28]. *Ae*. *aegypti* in Colombia has been found at altitudes up to 2,300 m above sea level [29]. The presence of *Ae*. *aegypti* across Colombia is mirrored by a high nationwide incidence of dengue, Zika and chikungunya. Dengue has been consistently reported in Colombia over the past two decades causing an average of 84,926 cases each year (1980–2019). Zika was first reported in Colombia in 2015 and was followed by a significant outbreak of 91,711 cases in 2016. Chikungunya was first detected in Colombia in 2013, causing 275,907 cases in that single year [30]. Colombia is now hyperendemic for dengue [31] as well as endemic for both Zika and chikungunya [32].

In addition to climatic variables, socio-economic (SE) factors can contribute to the spread of mosquito borne diseases. This is particularly acute with *Ae*. *aegypti*, a highly anthropophilic species which lives within or in close proximity to human dwellings breeding in domestic water storage containers. Poor housing construction together with high population density and inadequate sanitation with little to no access to clean running water are key SE factors promoting *Ae*. *aegypti* populations [33]. In the absence of suitable vaccines for dengue, Zika or chikungunya, disease prevention is currently based on *Ae*. *aegypti* control. This is challenging with a diurnal biting mosquito. A compound effect is the development of insecticide resistance in populations of *Ae*. *aegypti* reported in areas of Colombia [34].

This study aims to investigate the epidemiology of these three arboviruses (dengue, Zika and chikungunya) co-circulating in a single vector species (*Ae*. *aegypti*) in three distinct eco-systems in Colombia between 2007–2017. In addition to having differing climatic and socio-economic profiles the three locations selected as our study domain—Bello, Cúcuta and Moniquirá - have contrasting levels of *Ae*. *aegypti* circulation [35–38]. There is also a higher prevalence of insecticide resistance in *Ae*. *aegypti* from Cúcuta, specifically to common larvicides (i.e. temephos) used in vector control interventions for disease prevention [37]. The national vector interventions monitored by the National Insecticide Resistance Surveillance Network (Colombia’s National Institute of Health: INS) [39], following WHO recommendations (WHO) [40], continue to yield mixed local outcomes in control of mosquito populations and arboviral diseases [41–43]. We sought to assemble and model comprehensive sets of data for this recent 11-year period to investigate the climatological as well as the socio-demographic traits in these three locations that might correlate with disease prevalence–a proxy for levels of vector circulation. This is with the aim to elucidate the influencers from local ecosystems that could ultimately dictate the efficacy of vector and vector borne disease interventions. We used multifactorial approaches of several meteorological and socio-economic factors with disease incidence. We show that specific climatological factors are strong drivers for these arboviral diseases to which contextual socio-economical characteristics can act as modifiers. Importantly, we find a discriminatory pattern between these three diseases highlighting unexpected dynamics of transmission between Zika and dengue particularly in an area of high dengue circulation.

## Methods

### Study locations

**Fig 1:**
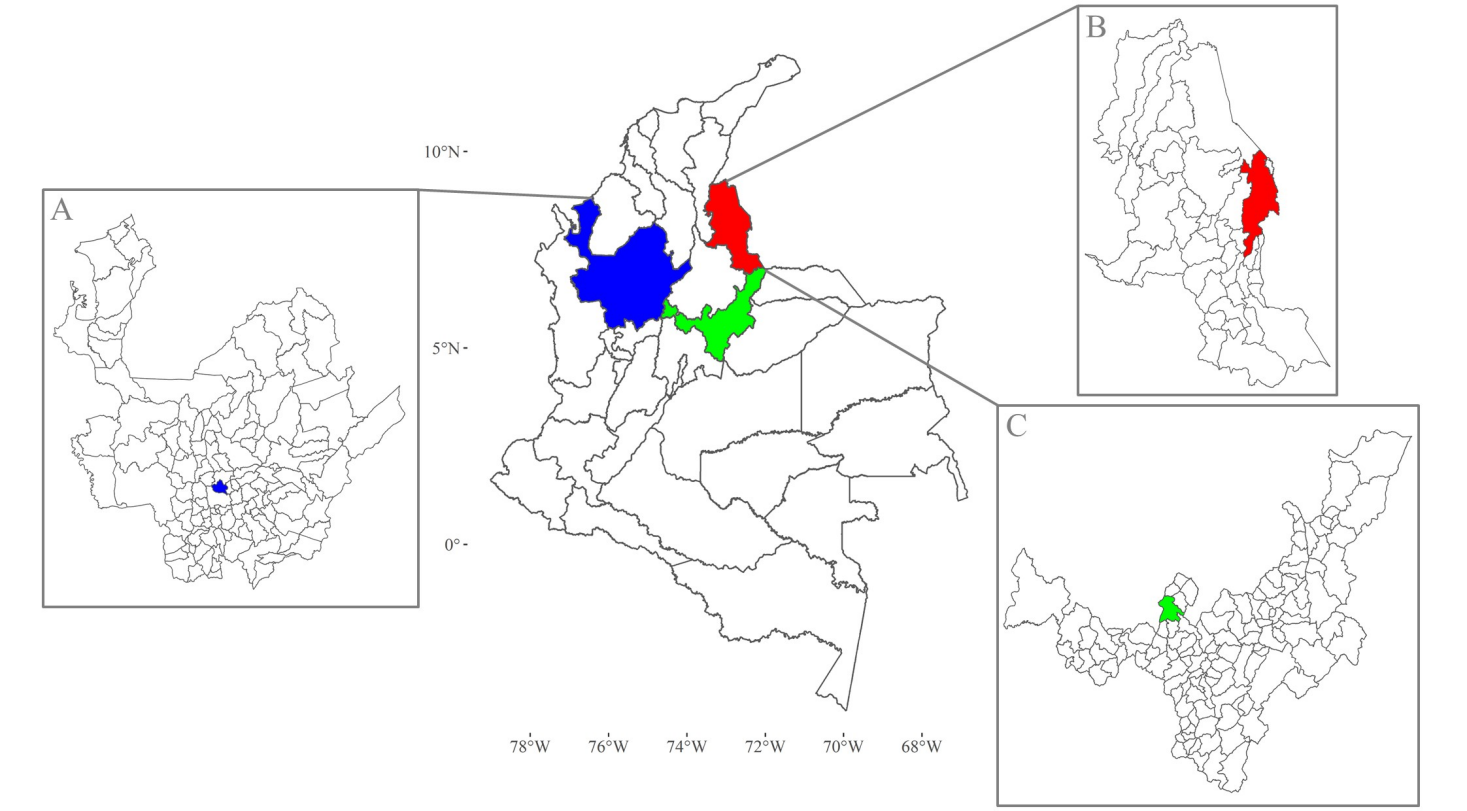
Map of Colombia showing the location of each municipality within their political divisions (departments). Departments are the largest units of local government answerable to the country’s national government. (A) Department of Antioquia governs Bello which is denoted as a small blue area. (B) Department of Norte de Santander has as its capital Cúcuta, a city (red) to the East of this department that shares the border with Venezuela. (C) Department of Boyacá has the municipality of Moniquirá (green). Map base layers were obtained from https://data.humdata.org/dataset/colombia-administrative-boundaries-levels-0-3 covered by a Creative Commons Attribution 4.0 International (CC BY) License (https://creativecommons.org/licenses/by/4.0/legalcode). Map base layers were modified by the addition of colours.

Three study municipalities: Bello, Cúcuta and Moniquirá (Fig 1) were chosen due to their geographical separation, and distinct climate characteristics, demographics and burden of *Ae*. *aegypti* borne diseases (Table 1).

**Table 1:** Climate, population and disease incidence for each municipality. Elevation, yearly precipitation, mean temperature and humidity were calculated from climatological data for 1981–2010 [44]. Population for each municipality calculated from 1985–2020 population projections (Source: National Administrative Department of Statistics: www.dane.gov.co [45]).There were increases in population size during the study period in Bello (388,401 in 2007 and 473,423 in 2017) and Cúcuta (599,905 in 2007 and 662,673 in 2017) but population remained relatively stable in Moniquirá (21,785 in 2007 and 21,284 in 2017). Disease incidence: Burden of *Ae*. *aegypti* borne diseases in cases per 100,000 people, calculated by taking number of cases and population in each year independently and combining these to give total incidence for all years (2007–2017) [46]. Elevation in metres (m), precipitation in millimetres (mm) and temperature in degrees Celsius (^o^C).

Municipality	Elevation (m)	Annual Precipitation (mm)	Mean Temperature (^o^C)	Mean Relative Humidity (%)	Climate Classification (Caldas-Lang)	Population	Disease Incidence (per 100,000)
**Bello**	1438	1542.44	21.96	76.80	Temperate Semi-Wet	356,504	1301
**Cúcuta**	250	904.18	27.19	71.05	Warm Semiarid	558,599	6094
**Moniquirá**	1700	2006.25	19.09	76.53	Cold Humid	21,249	4669

### Disease incidence data

Cases of dengue, severe dengue (dengue shock syndrome and dengue haemorrhagic fever), chikungunya and Zika reported in each epidemiological week were obtained for the period of 2007 to 2017 from SIVIGILIA (National Public Health Surveillance System, Colombian National Institute of Health) [46]. This period of study (2007–2017) had consistent reporting of dengue along with the epidemics of both Zika and chikungunya. Chikungunya was first reported in 2014 and Zika in 2015. For the purpose of analysis week 53 was removed from any years in which there were 53 epidemiological weeks (2008 and 2014) this ensured continuity across the data set with all years comprising of 52 weeks when analysed. Only cases confirmed by SIVIGILIA were used in this analysis, confirmations are made based on laboratory tests and epidemiological links. The disease incidence data used in this study is provided in detail in S1 Table.

### Climate data

Daily weather data for Bello, Cúcuta and Moniquirá were obtained from the NASA Langley Research Center (LaRC) POWER Project [47]. The meteorological data has a resolution of 0.5^o^ latitude and 0.5^o^ longitude, with data taken from 6.3367^o^ latitude and -75.5596^o^ long for Bello, 7.8891^o^ latitude and -72.4967^o^ for Cúcuta and 5.879^o^ latitude and -73.5736^o^ longitude for Moniquirá. The weather data was obtained for the same time period as the disease incidence data (2007–2017) and converted to epidemiological weeks to correspond with the dates of the disease incidence data. The weather variables included for each municipality were: maximum temperature (Tmax), minimum temperature (Tmin), average temperature (Tavg), maximum wind speed (WSmax), minimum wind speed (WSmin), average wind speed (WSavg), total precipitation and average humidity (Havg). The climate data used in this study is provided in detail in S1 Table.

### Population and socio-economic data

Population data for each municipality were obtained using population projections by Colombia’s National Administrative Department of Statistics (Departamento Administrativo Nacional de Estadística) (DANE) (www.dane.gov.co) [45]. The multidimensional poverty index (MPI) was implemented by the Oxford Poverty and Human Development Initiative and the United Nations Development Program’s Human Development Report Office as a direct method for measuring poverty [48]. The MPI at municipality level was obtained from DANE using data collected in the 2018 National Population and Housing Census and using the indicators and respective weightings listed in S2 Table [49]. The overall multidimensional poverty index for each study municipality is shown in Table 2 along with the values for each socio-economic indicator in each municipality. The socioeconomic variables included in the MPI calculation and their specific interpretation within Colombia are explained in S2 Table. The overall MPI calculations can be interpreted as higher values indicating higher proportion of the population in poverty. Households with values of > 33.3% in any indicators are classed as poor.

**Table 2:** Socio-economic variables for the three municipalities of Bello, Cúcuta and Moniquirá overall and for each individual indicator. The socioeconomic variables included in the MPI calculation and their specific interpretation within Colombia are explained in S2 Table. The overall MPI calculations can be interpreted as higher values indicating higher proportion of the population in poverty.

Indicator	Total by Municipality (%)		
	Bello	Cúcuta	Moniquirá
Socio-economic Status			
Multidimensional Poverty Measure ^(a)^	14.2	25.7	27.1
Education			
Illiteracy	4.4	8.4	15.3
Low Educational Achievement	36.5	46.7	59.4
Childhood and Youth			
School lag/failure	13.2	17	11.1
School absence	2.8	4.6	3.8
Barriers to early childhood services	1.8	2.2	1.6
Child labour	0.5	1.1	1.2
Health			
No health insurance	19.2	17.5	12.4
Barriers to health services	2.8	5.2	2.9
Employment			
Informal work	72.7	87	85.7
Dependency rate	25.4	34.3	29.2
Housing Conditions			
No access to improved water	5.6	3.9	20.4
Inadequate excreta disposal	5.5	5.8	12.1
Inappropriate flooring material	0.7	3.6	4.3
Inappropriate wall exterior	1.4	6.1	0.6
Critical overcrowding	5.6	16.4	4.7

(a) Calculated using the data in S2 Table. Households with values of > 33.3% in any indicators are classed as poor.

### Statistical analysis

#### Patterns of disease incidence by location

Differences in the total burden of all three *Ae*. *aegypti* borne viruses as well as the individual burden of dengue and severe dengue were investigated using the total number of cases from 2007–2017. Poisson Generalised Linear Models (GLMs) were initially carried out because they allow for examination of non-linear data with response variables that are not normally distributed [50], revealing overdispersion (data variance greater than expected for the given model) statistics of 545, 542 and 123 for total disease, dengue and severe dengue respectively. To correct for the large overdispersion the GLMs were recalculated with negative binomial errors [51] using the glm.nb function of the R package MASS [52]. Differences between all three municipalities were tested with Tukey pairwise comparisons using the glht function of the multcomp R package [53]. Incidence of chikungunya in 2015 was initially modelled using a Poisson GLM which revealed an overdispersion statistic of 2.9. The standard errors were therefore corrected using quasi-GLMs where the variance was theta x mu. Where mu was the mean of the dependant variable and theta the dispersion parameter of the quasi-GLMs [51]. Quasi-GLMs were conducted using the glm function from the R package stats [54]. Zika incidence was modelled for the year 2017 only, initially a Poisson GLM was used and an overdispersion statistic of 99 was detected. As the overdispersion statistic was above 20 it was corrected for using negative binomial errors [51]. Total *Ae*. *aegypti* borne disease and dengue incidence were also modelled for 2015 and 2016 using quasi-GLMs to account for low level overdispersion except for total disease in 2016 which had a dispersion statistic of 90 and was therefore modelled with a negative binomial GLM. Population was used as an offset in all models in order to standardise disease incidence by population size.

#### Patterns of disease incidence over time

For the pattern of disease over time we used total yearly incidence data. Poisson GLMs were initially used to model each disease in each municipality but overdispersion was detected in some models, hence error distributions were adjusted accordingly. For Bello incidence of both total disease (dengue, severe dengue, chikungunya and Zika) and dengue alone were modelled using quasipoisson GLMs, correcting for overdispersions of 3.69 and 3.63 respectively. For Cúcuta a negative binomial GLM was required for total disease incidence in order to correct for overdispersion of 32.52 and quasi-GLM was used for dengue incidence due to slight overdispersion of 4.87. Severe dengue incidence in Cúcuta was not found to be significantly overdispersed when modelled with a Poisson GLM (overdispersion statistic = 1.79), as the overdispersion statistic was <2. For Moniquirá both total disease incidence and dengue incidence alone were modelled using quasi-GLMs, correcting for respective overdispersion statistics of 2.39 and 2.34 [51]. Population was used as an offset in all models in order to standardise disease incidence by population size. All quasi-GLMs were conducted using the glm function from the R package stats [54] and negative binomial GLMs used the glm.nb function of the R package MASS [52]. Differences between the years were tested with Tukey pairwise comparisons using the glht function of the multcomp R package [53].

#### Patterns of climate over time and between municipalities

Patterns in climate over time in each municipality were visualised by fitting the data with a non-parametric approach (locally weighted scatterplot smoothing) as implemented in R’s ggplot2 loess (local polynomial regression fitting) method. Local fitting uses the distance of data in the neighbourhood of each dependent variable (time) to weight the least squares of the independent variable (climate variable quantitative measurement). The size of the neighbourhood is controlled by the ggplot’s span parameter in geom_smooth or stat_smooth. The default span applied here uses tricubic weighting (proportional to (1 − (distance/max distance)^3)^3) [55].Tukey’s Honest Significant difference method was used following an ANOVA to test for differences in each climate variable between the study municipalities using the R package stats [54].

#### Generalised additive models

The correlations between climatic variables and the total disease incidence (dengue, severe dengue, chikungunya and Zika) across all three locations were investigated using a generalised additive model (GAM). The weekly disease incidence and weather data for each municipality was converted into 4-week data, matching the dates of epidemiological months. Combining the data into 4-week periods rather than individual weeks reduced zero inflation improving the reliability of the GAM outputs. All climate variables were lagged by plausible time lags for their effect on disease incidence, of 4 and 8 weeks. Square root transformations were used for total disease incidence and each weather variable due to non-normal distribution. Generalised additive models were chosen due to their ability to model non-linear relationships between a response variable (disease incidence) and multiple explanatory variables (climate variables) [56]. A quasi-maximum likelihood Poisson GAM was used in order to prevent possible overdispersion [51]. Population size was used as an offset to standardise disease incidence by population. Initially all climate variables with both 4 and 8-week time lags were assumed to have a non-linear relationship and were therefore modelled as smoothed terms. Subsequent analysis of the effective degrees of freedom (*edf*) was used to identify variables with *edf* = 1.0, suggesting linearity. These variables were then included in the model as linear rather than smoothed terms. Generalised cross validation (GCV) was used to determine the most appropriate model. Generalised additive modelling and subsequent model validation was conducted using the R package mgcv [57]. Visualisation of GAM estimations were conducted using the mgcViz R package [58].

#### Socio-economic factors

Principle components analysis (PCA) was used for dimensional reduction to allow the inclusion of socio-economic data with previously compiled geographic and climate data. The PCA was conducted using the R package stats [54] and visualisations of the PCA were created using the factoextra R package [59].

## Results

### General disease incidence between 2007–2017

**Fig 2:**
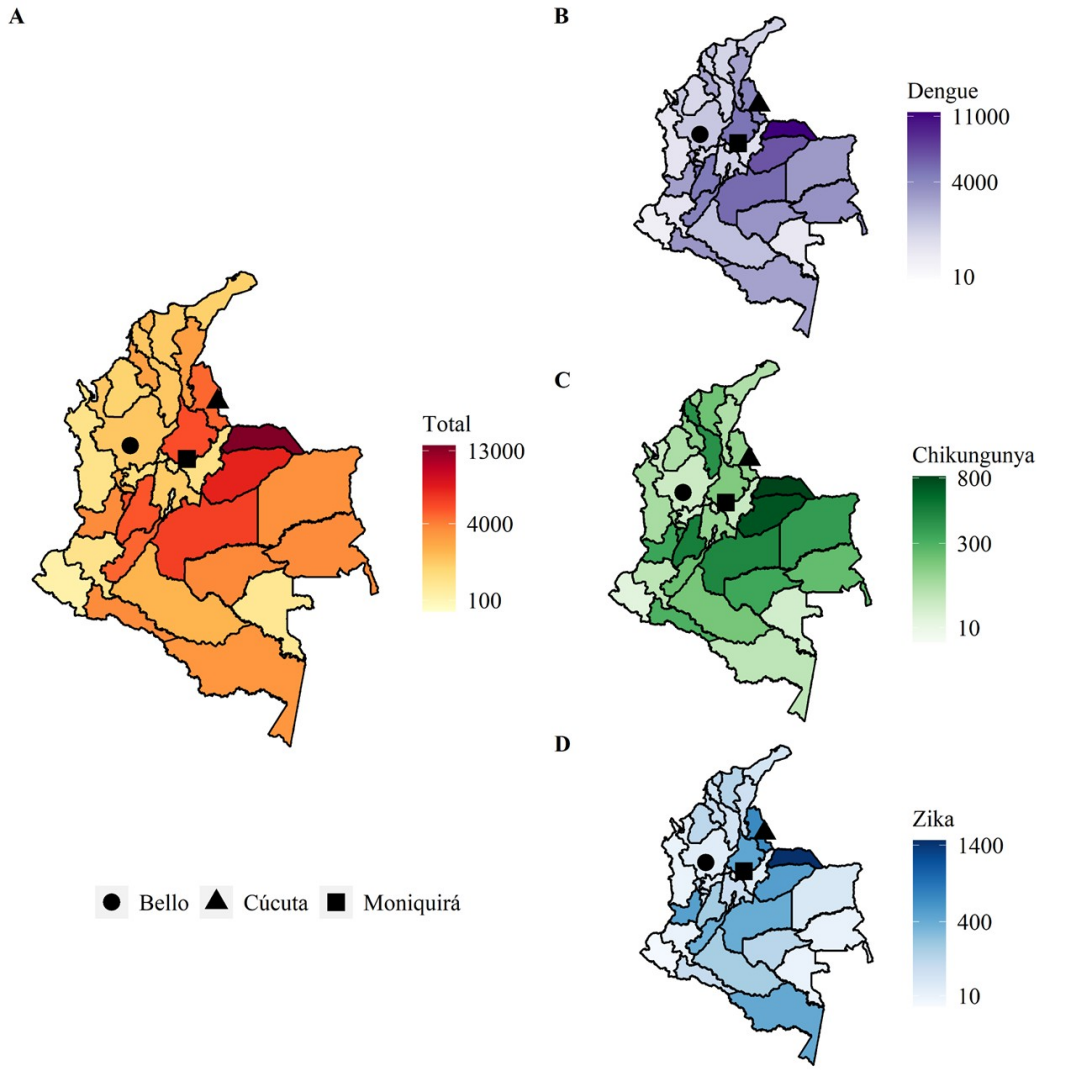
Arboviral diseases in Colombia per 100,000 population for the period of 2007–2017. (A) Number of total *Ae*. *aegypti* borne diseases in Colombia. (B) Number of cases of dengue, (C) chikungunya and (D) Zika. Map base layers were obtained from https://data.humdata.org/dataset/colombia-administrative-boundaries-levels-0-3 covered by a Creative Commons Attribution 4.0 International (CC BY) License (https://creativecommons.org/licenses/by/4.0/legalcode). Map base layers were modified by the addition of colours.

Dengue was the most prevalent of the three diseases throughout Colombia (Fig 2 and S3 Table). Cúcuta carried the highest disease burden of the three municipalities followed by Moniquirá, with Bello having the lowest disease incidence. The total number of confirmed cases of all *Ae*. *aegypti* borne diseases (dengue, severe dengue, chikungunya and Zika) during the period of 2007–2017 were 5,727, 32,328 and 1,005 in Bello, Cúcuta and Moniquirá respectively. The breakdown of cases per 100,000 people of all three diseases between 2007 and 2017 in these locations were 1,301, 6,094 and 4,669 in Bello, Cúcuta and Moniquirá, respectively. When discriminating by disease per 100,000 people, the incidence of dengue was 1,263 in Bello, 5,106 in Cúcuta and 4,566 in Moniquirá. Chikungunya had 27 cases in Bello, 154 in Cúcuta and 61 Moniquirá. Incidence of Zika was lowest in Bello with 11 cases compared to 834 in Cúcuta and 42 in Moniquirá.

**Fig 3:**
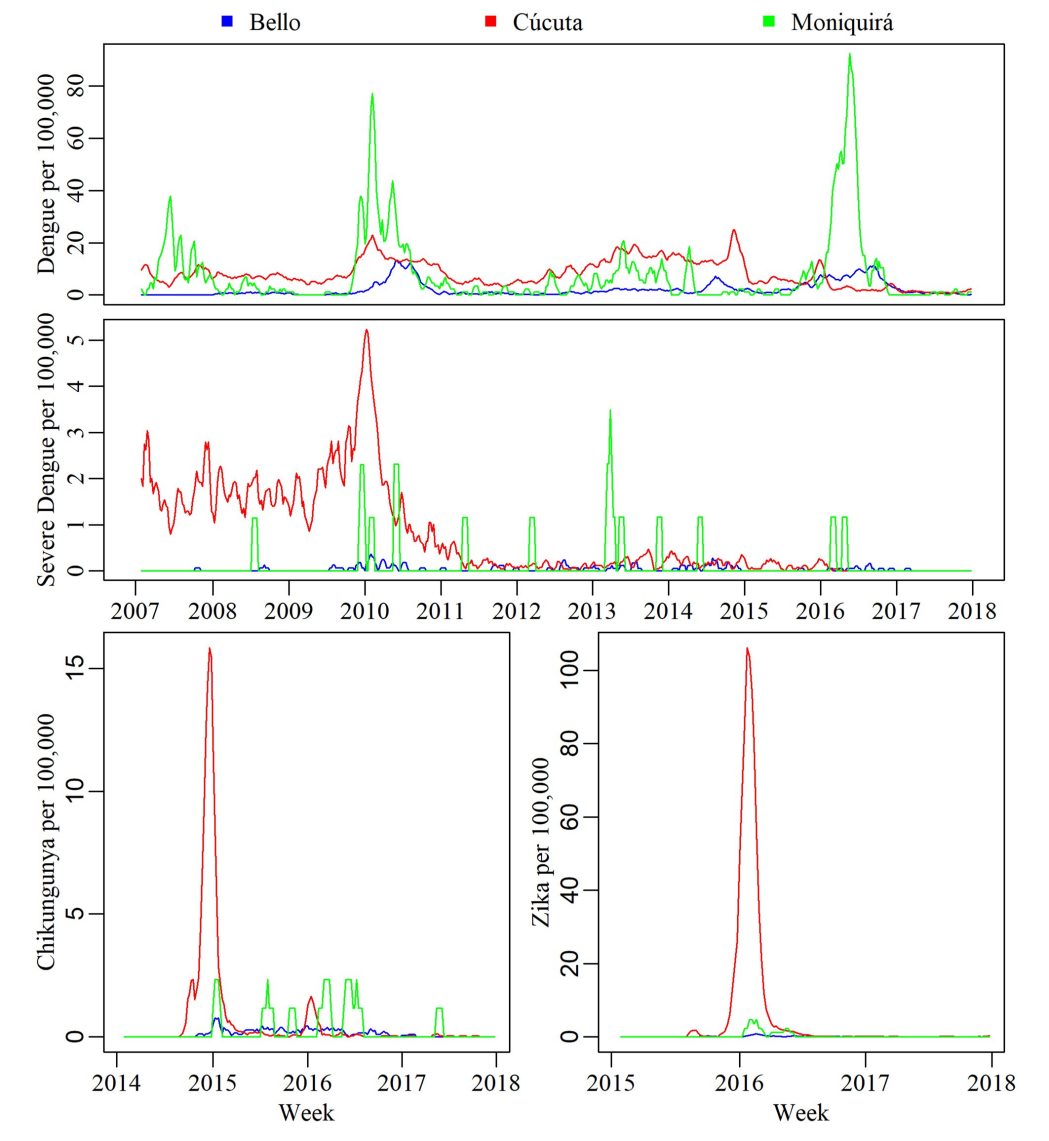
Data granularity for disease reported from 2007–2017. Smooth moving averages (SMA) 4-week time series of dengue (2007–2017), severe dengue (2007–2017), chikungunya (2014–2017) and Zika (2015–2017) cases per 100,000 people in Bello, Cúcuta and Moniquirá.

For visualisation of the spread of disease data over the study period, 4-week smooth moving averages (SMA) were applied (Fig 3). SMA were only used for data visualisation (Fig 3) and not during the analysis of the disease data. Dengue and severe dengue were consistently reported throughout the period of 2007–2017. Dengue data showed at least three spikes: one in 2009 (Fig 3), followed by two more by the end of 2014 and beginning of 2017.The latter of these peaks started approximately two years prior (2015) (Fig 3). Chikungunya cases were only reported between 2014–2017, hence Fig 3 shows chikungunya cases for this time period only. This was similar for Zika, whose first cases were reported in 2015, with the highest number of cases reported in week 468 in 2016 (Fig 3). There were no obvious seasonal patterns in disease incidence reported across the three municipalities (Fig 3), due to the close location of the study municipalities to the Equator. However the major peaks depicting the chikungunya (2015) and Zika (2016) outbreaks in Cúcuta (2015) both occurred within the early part of the year, with cases beginning to increase from the last few months of the previous year, this is also true of the spike in severe dengue in Cúcuta in 2010. Whilst Colombia is an Equatorial country and does not experience distinctive seasonal patterns in climate incidence, the spike in severe dengue in Cúcuta in 2010 corresponds to higher than average temperatures as identified by analyses of meteorological characteristics conducted in this paper. The timings of the chikungunya and Zika peaks do not however correspond to any deviations in meteorological characteristic averages and are more likely due to the timing of importation of the viruses into the local areas.

### Patterns of disease incidence per location

**Fig 4:**
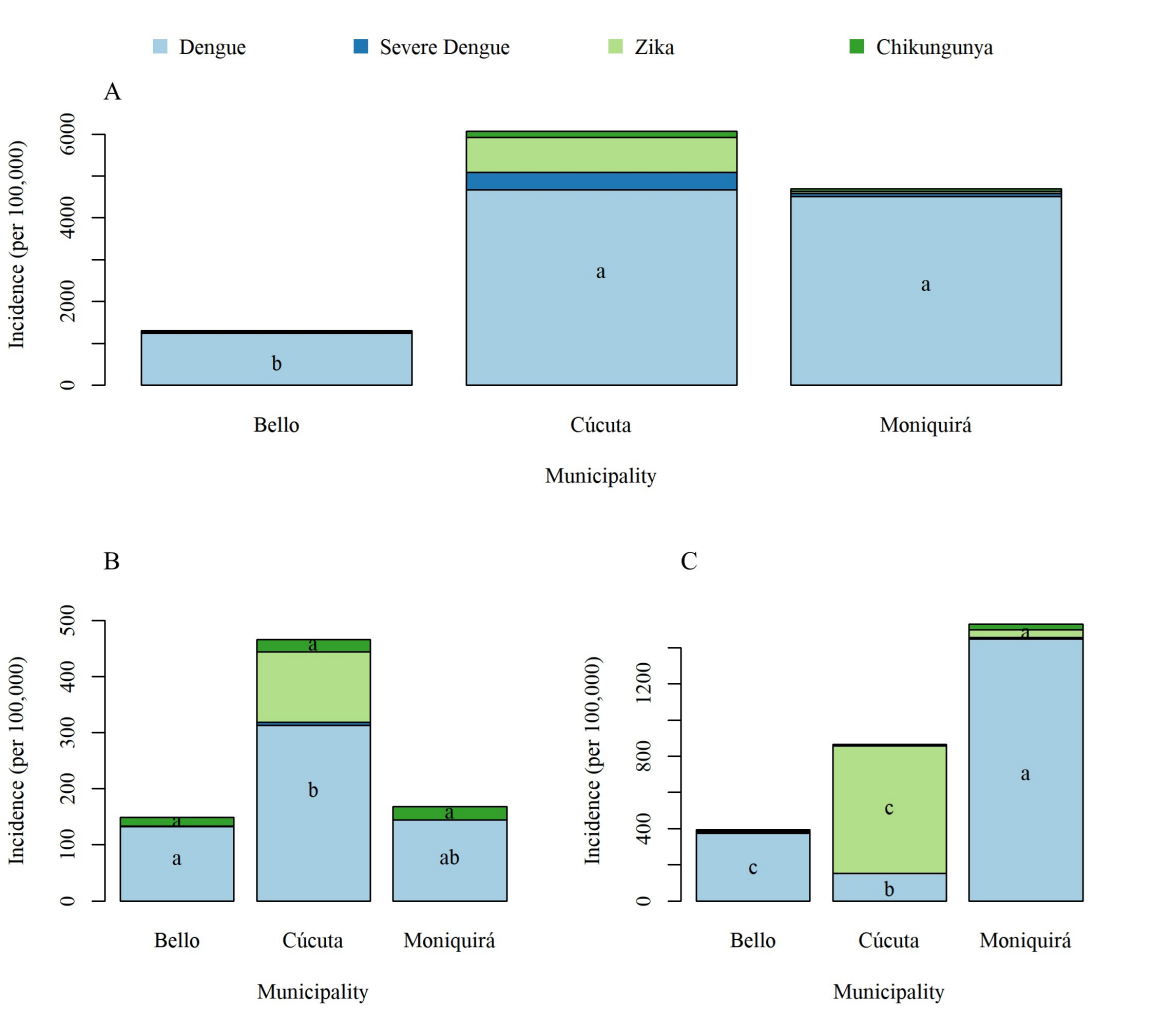
Total number of cases of dengue, severe dengue, chikungunya and Zika per 100,000 in the three municipalities. (A) 2007–2017, (B) 2015 and (C) 2016. The letters indicate significance of post-hoc Tukey test, where letters are different this indicates a significant difference (p = < 0.05).

Total disease incidence over the 11-year period was significantly lower in Bello than in Cúcuta (p = < 0.001) and Moniquirá (p = 0.005) (Fig 4A) for all three diseases. The number of dengue cases were similarly high between Moniquirá and Cúcuta (*p* = 0.99). However, severe dengue incidence was significantly different across all three municipalities: Cúcuta had the highest burden of severe dengue when compared to both Bello (p = <0.001) and Moniquirá (p = 0.005), and Moniquirá had a significantly higher burden of severe dengue when compared to Bello (p = 0.047) (Fig 4A).

The incidence data were also analysed separately for the years when the outbreaks of chikungunya and Zika were reported, 2015 and 2016, respectively (Fig 4B and 4C). This allowed for a more directly and meaningful comparison of the burden represented by these three diseases. Cases of chikungunya in 2015 were not significantly different between any of the municipalities (Fig 4B). However, Cúcuta had significantly higher incidence of Zika than both Bello (p = <0.001) and Moniquirá (p = <0.001) (Fig 4C). Interestingly, in the same year of the Zika outbreak (2016) each municipality had a significantly different number of dengue cases. Cúcuta had the lowest incidence of dengue, and Moniquirá the highest (Fig 4C). While Cúcuta had the highest incidence of dengue in previous years, in 2016 the same location experienced the lowest incidence of dengue accompanied by the highest incidence of Zika (Fig 4C).

### Patterns of disease incidence during 2007 to 2017

**Fig 5:**
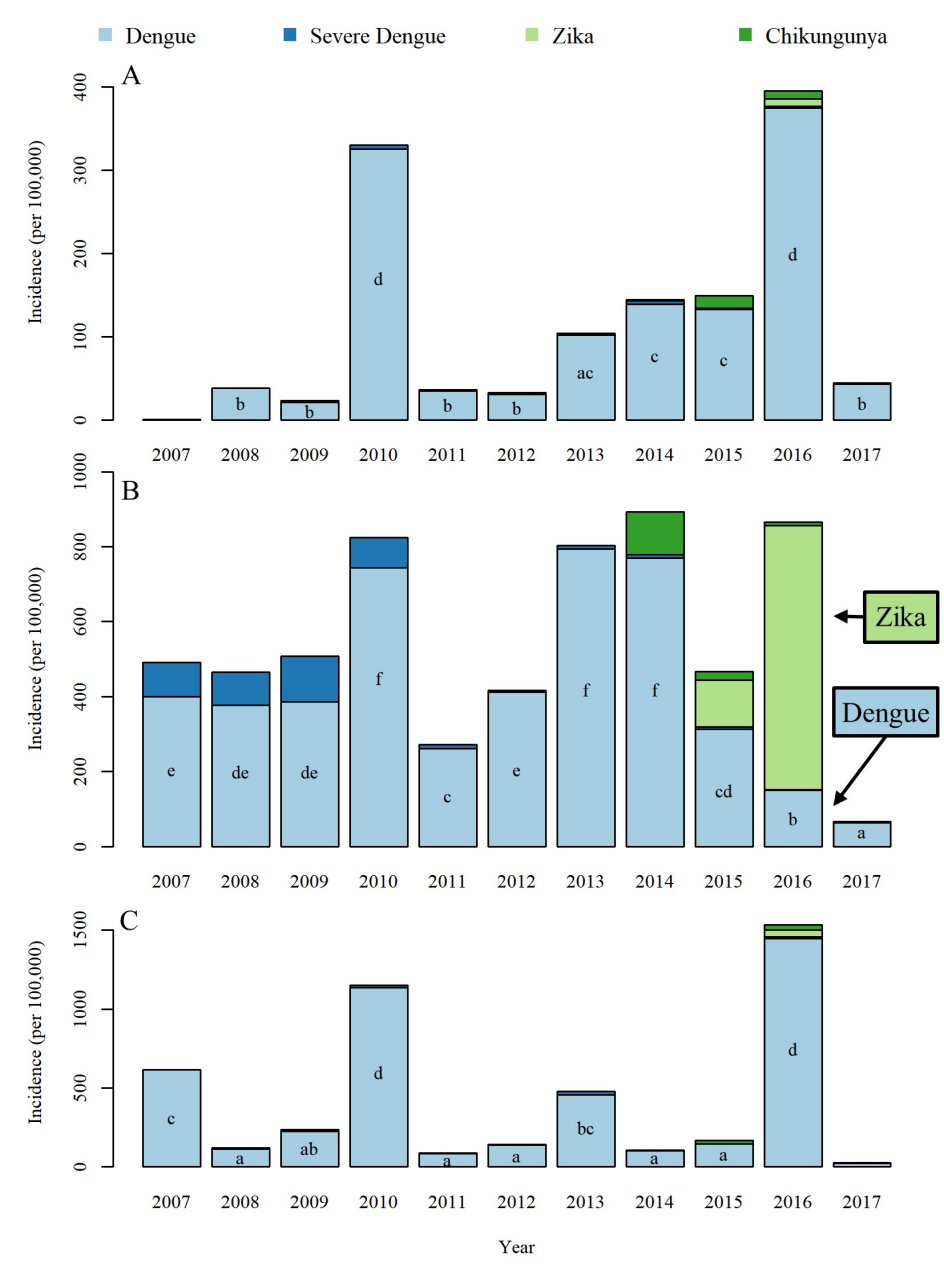
Annual cases of dengue, severe dengue, chikungunya and Zika per 100,000 people. (A) Bello, (B) Cúcuta and (C) Moniquirá. The letters indicate significance of post-hoc Tukey test, where letters are different this indicates a significant difference (p = < 0.05). Post-hoc Tukey tests show differences between years within each municipality. Note the spike in cases of dengue in 2010 in all three locations and the opposite trend in cases of Zika and dengue for Cúcuta (B) between 2015 and 2016.

We compared the number of cases per 100,000 people in each year for dengue, severe dengue, chikungunya and Zika from 2007 to 2017 in Bello, Cúcuta and Moniquirá (Fig 5). The initial cases of Zika were first confirmed in Colombia in 2015, with cases reported in both Bello and Cúcuta from that year onwards. The first Zika case in Moniquirá was not confirmed until 2016. Whilst cases of Zika were relatively low in Bello and Moniquirá, Cúcuta experienced large outbreaks (Fig 5).

The data analysed per location (Fig 4) and per year (Fig 5) suggested that Zika displaced dengue in Cúcuta from 2015 to 2016. The number of Zika cases per 100,000 population in 2015 were 125 and 705 in 2016 whilst dengue was present in 313 and 150 cases, respectively (Fig 5B). On the other hand, in Bello and Moniquirá, where the incidence of Zika was much lower (Bello; 1 in 2015 and 3 in 2016, Moniquirá; 0 in 2015 and 42 in 2016), there was an incremental trend for dengue during this same transition from 2015 to 2016 (Fig 5A and 5C). Following the significantly high dengue incidence in Bello and Moniquirá in 2016, the incidence stabilised, and the incidence reported in 2017 were not statistically different to that of years prior to 2016. This was not the case however in Cúcuta where incidence of dengue continued to fall, with the lowest incidence of the study period observed in 2017 (Fig 5B). In 2017 incidence of Zika was also much lower with only 3 cases per 100,000 people reported in Cúcuta in 2017 (Fig 5A and 5C).

### Effect of climate on disease incidence

**Fig 6:**
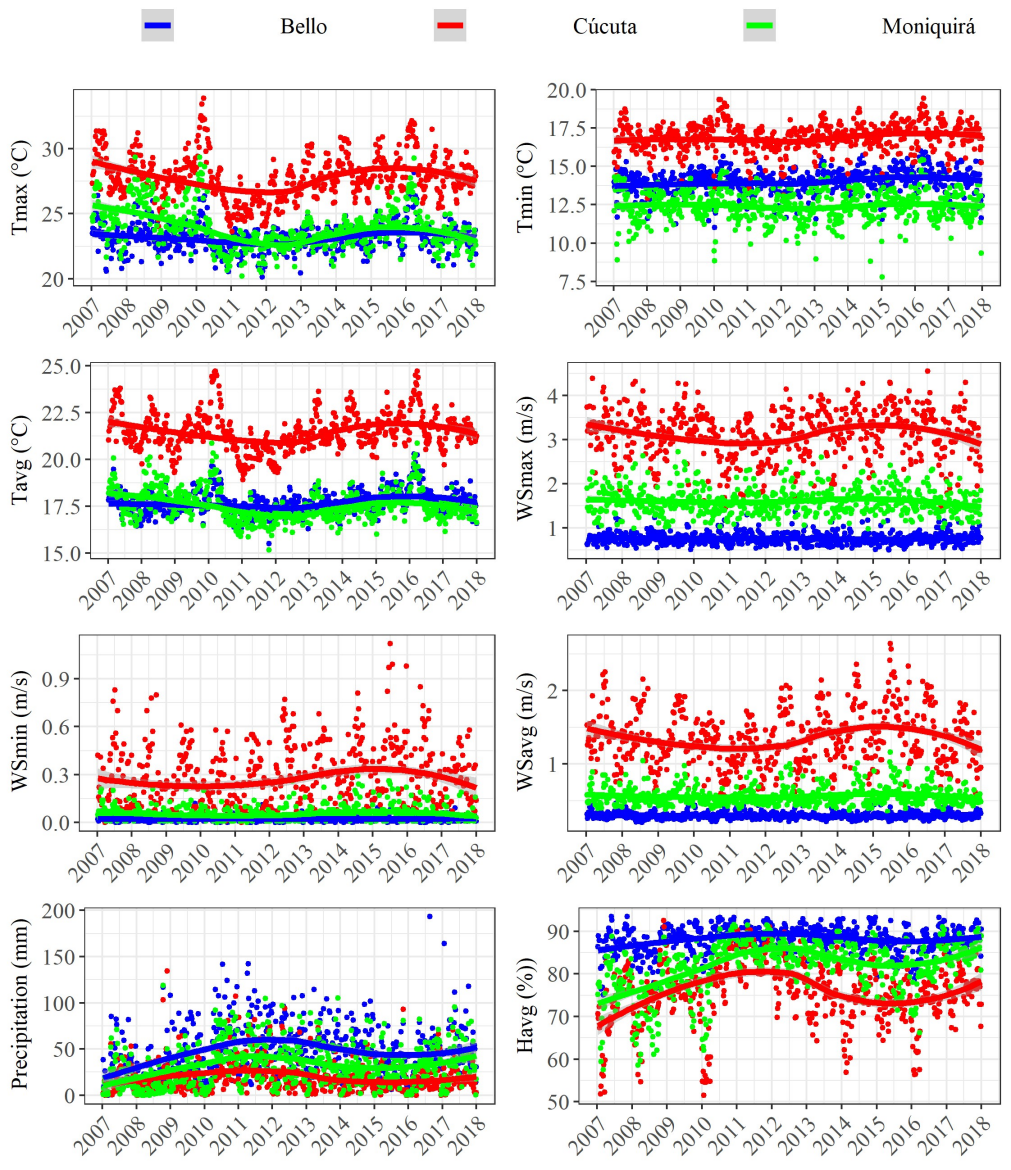
Data for climate in the municipalities of Bello, Cúcuta and Moniquirá between 2007 and 2017. Maximum temperature (Tmax), minimum temperature (Tmin), average temperature (Tavg), maximum wind speed (WSmax), minimum wind speed (WSmin), average wind speed (WSavg), precipitation and average relative humidity (Havg), for Bello, Cúcuta and Moniquirá between 2007 and 2017 fitted using LOESS.

The geographical settings for the three locations studied here Bello, Cúcuta and Moniquirá (Table 1) determine three different climate systems–ecosystems. These three different ecosystems are expected to establish contrasting behavioural patterns for the mosquito vector species that transmit dengue, Zika and chikungunya. The results presented in this section related to the quantitative climate variables as explained in Methods and inferred here to be proxy determinants of disease transmission by *Ae*. *aegypti*. We initially summarised the climate variables of each municipality from 2007–2017 using LOESS to show the patterns over time (Fig 6). All parameters used here for temperature (Tmax, Tmin, Tavg) and wind speed (WSmax, WSmin, WSavg) were highest in Cúcuta. Bello had the lowest wind speed and highest relative humidity (Havg) and precipitation. There was a large fluctuation of 8°C in Tmax and 5°C in Tavg in 2010 in all three locations (Fig 6). Otherwise the recorded climatic variables fluctuate within a similar range throughout these 11 years. The variability between municipalities was greater than within and therefore granted the GAMs as applied in this study. We found that the climate experienced by the three municipalities was significantly different (p = <0.001) over this 11-year period for all the climate parameters included in this study (S4 Table).

We explored potential relationships between the time series climate data and total disease incidence in Bello, Cúcuta and Moniquirá at shorter 4 and 8-week time lags using a generalised additive model (GAM) as explained in Methods. The estimates from the quasipoisson GAM explained 57.6% of the variance in total disease incidence over time (Table 3). Effective degrees of freedom (*edf*) close to 1 represent relation close to linearity while high *edf* values for the smooth terms suggest that the relationship between climatic variables and disease incidence is non-linear. The GAM identified significant relationships between disease incidence and precipitation at 4 and 8-week lags, average humidity (4-week lag), minimum temperature (4 and 8-week lags), average temperature (8-week lag), maximum wind speed (4 and 8-week lag) and average wind speed (4-week lag) (Table 3).

**Table 3:** Quasi-GAM model estimates of the effects of climate variables on total disease incidence in Bello, Cúcuta and Moniquirá. Climate variables: maximum temperature (Tmax), minimum temperature (Tmin), average temperature (Tavg), maximum wind speed (WSmax), minimum wind speed (WSmin), average wind speed (WSavg), total precipitation and average humidity (Havg) with 4 (lag4) and 8 (lag8) week time lags. The model statistics, GCV is the minimised generalised cross validation which was used for smoothness selection. Explained variance is the percentage of total variance the Quasi-GAM model could explain. For smooth terms the effective degrees of freedom *(edf*) and F-statistic (F). For linear terms the slope estimate (Estimate) and standard error of the mean (SE). (*) Significant variable at the 0.001 level.

Model Statistics	GCV	2.0692	
	Explained Variance	57.6%	
Smooth Terms	**Variable**	***edf***	**F**
	Tavg_lag4	6.589	1.535
	Pre_lag4*	3.801	5.684
	Havg_lag4*	7.472	2.574
	WSmax_lag4*	6.734	6.377
	Tmin_lag8*	1.536	5.98
	Tavg_lag8*	2.97	4.896
	Pre_lag8*	1.918	7.245
	WSmax_lag8*	5.63	2.875
Linear Terms	**Variable**	**Estimate**	**SE**
	Tmax_lag4	-0.1931	0.5801
	Tmin_lag4*	-0.9657	0.3596
	WSmin_lag4	0.2055	0.3009
	WSavg_lag4*	-1.5992	0.6095
	Tmax_lag8	0.6437	0.4817
	Havg_lag8	0.311	0.536
	WSmin_lag8	-0.26	0.3008
	WSavg_lag8	0.9054	0.5907

**Fig 7:**
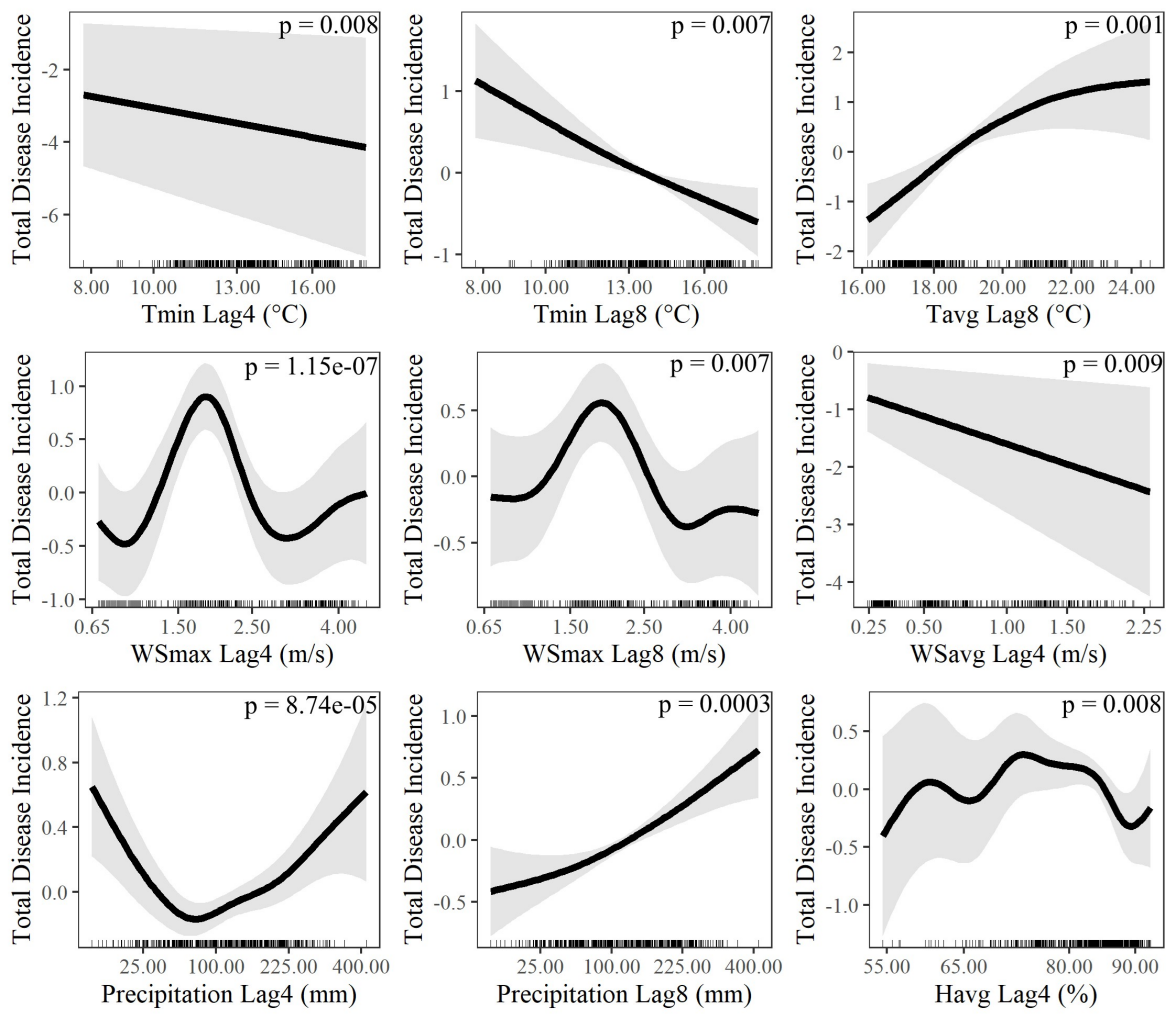
Climate determinants of total disease incidence. GAM estimated relationships (solid black line) and corresponding 95% confidence limits (grey shaded area) between relative disease risk and minimum temperature (Tmin) with 4 (A) and 8-week (B) time lags, average temperature (Tavg) with 8-week time lag (C), maximum wind speed (WSmax) with 4 (D) and 8-week (E) time lags, precipitation with 4 (G) and 8-week (H) time lags and average humidity (Havg) with 4 week time lag (I). P values for each relationship are also shown.

The climate variables significantly correlated to total disease incidence as presented in Table 3 were further investigated by plotting the smoothed variance of the latter against the ranges covered for each climate variable (Fig 7). The analysis of the three most significant climatic contributors to total disease incidence -temperature, wind speed, and precipitation–delineated clear trends on how climate affected disease transmission. Increasing Tmin from 8°C to 16°C, at either 4 or 8-week, reduced the total disease incidence while the average temperatures (Tavg) up to 24°C contributed to incremental levels of disease (Fig 7A–7C). This could be an indication of the need for fluctuations at low temperatures in order to facilitate increased virus transmission.

Increasing wind speed above 1 m/s was associated by an increase in disease incidence with a peak around 2 m/s after which disease incidence declined until around 3 m/s where a small rise in disease risk can also be seen. This relationship between maximum wind speed and disease incidence was observed after both 4 and 8-week time lags (Fig 7D and 7E). The decisive influence on wind speed was substantiated by the negative effect on disease incidence at incremental average wind speeds (Fig 7F). Precipitation showed a positive relationship with disease incidence above 90 mm at 4-week time lags and above 25 mm for the data with 8-week time lag (Fig 7G–7H). The high level of non-linearity shown for the relationship between average humidity and disease incidence (*edf* = 7.5) (Table 3) is detailed in Fig 7I. Disease incidence increased as humidity increased between 55–60%. This was followed by a slight decrease and plateau between 60–70%, a more rapid increase was observed between 70 and 75% above which disease incidence begins to decline (Fig 7I).

### Socio-economic profiles

We followed a holistic approach by further including socio-economic data for these three locations in the investigation for modifiers to the disease transmission of dengue, Zika and chikungunya. The overall multidimensional poverty index (see Methods) was lowest in Bello at 14.2. Cúcuta and Moniquirá had similar MPIs at 25.7 and 27.1 respectively (Table 2).

The overall MPIs in Cúcuta and Moniquirá were similar. However, there were differences in specific poverty measures relevant to the transmission of vector borne viral diseases. Cúcuta had higher rates of overcrowding (16.4%), barriers to both childhood and youth services (2.2%) and healthcare services (5.2%) and inappropriate exterior wall material (6.1%) (Table 2). The findings also pointed to other socio-economic indexes that also affect general health and well-being in Moniquirá: inadequate excreta disposal (sanitation) (12.1%), no access to an improved water source (20.4%), illiteracy (15.3%) and low education achievement (59.4%) were all highest in Moniquirá (Table 2).

**Fig 8:**
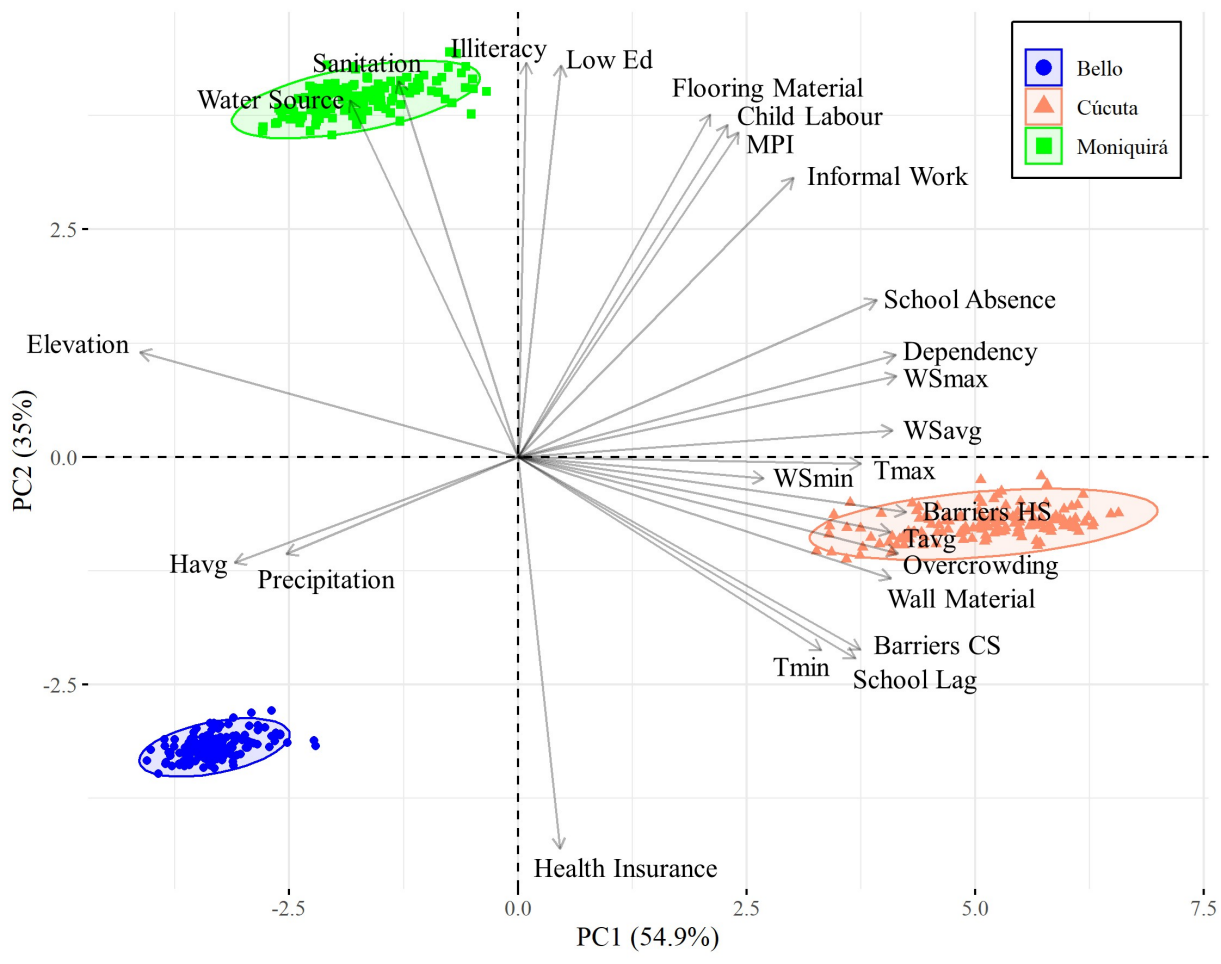
Principal component analysis for the both socio-economic and climate variables. Socio-economic variables: no access to improved water source (Water_Source), inadequate disposal for excreta (Sanitation), illiteracy, low educational achievement (Low_Ed), inappropriate flooring material (Flooring_Material), child labour, multidimensional poverty index (MPI), informal work, school absence, dependency rate (Dependency), barriers to health services (Barriers_HS), critical overcrowding, inappropriate wall material (Wall_Material), barriers to early childhood services (Bariers_CS), no health insurance (Health_Insurance). Climate variables: elevation, maximum wind speed (WSmax), average wind speed (WSavg), maximum temperature (Tmax), minimum wind speed (WSmin), average temperature (Tavg), precipitation (Pre) and average humidity (Havg). The length of the arrows represents the contribution of each variable.

In order to introduce the socio-economic data into the analyses undertaken with the epidemiological and climatic data we carried out a dimensionality reduction and correlation with a principal component analysis (PCA). Importantly there was a clear separation of the three municipalities along both dimensions PC1 and PC2 that together integrates 89.9% of the compiled parameters (Fig 8). This approach made apparent a discriminatory set of factors both climatic and socio-economic for all three locations. Cúcuta had an extensive combination of climate factors (i.e. wind speed and temperature) that together with school absence, dependency, overcrowding, wall material and school failure are potential modifiers of dengue, Zika and chikungunya risk in this municipality. Interestingly, Moniquirá showed mainly socio-economic variables (i.e. water source, sanitation, illiteracy, low education, flooring material, child labour, high MPI, informal work) to be potential modifiers for disease risk. On the other hand, Bello had mainly climate variables as potential modifiers of disease transmission–average humidity, precipitation and elevation–with only health insurance as a socio-economic factor.

## Discussion

This study set out to determine the longitudinal dynamics of three *Aedes* arboviral diseases co-circulating in three regions of Colombia over a 11-year period between 2007 and 2017. We found significant differences in the burden of viruses among the three municipalities studied. Bello had the lowest level of disease incidence across all diseases. Cúcuta had the highest incidence of severe dengue (2007–2017) and Zika (2016) and the highest overall disease incidence. In addition to climatic factors the burden of these vector borne diseases in Cúcuta can be compounded by local current socio-political dynamics. Cúcuta is on Colombia’s border with Venezuela, a country which has faced an economic, political and health crisis in recent years [60,61]. The humanitarian crisis has led to large migration of Venezuelan citizens and refugees to neighbouring countries, with Colombia receiving the highest number of Venezuelan migrants. The number of Venezuelan migrants in Colombia increased from 48,714 in 2015 to 600,000 in 2017 [62]. This has had a significant impact on public health in Colombia, with infectious and vector-borne diseases particularly effected [63–68]. The recent COVID-19 pandemic has intensified the humanitarian crisis in Venezuela, with bordering countries including Colombia closing their borders. The International Rescue Committee have reported a crisis for Venezuelan migrants in Cúcuta due to reduced access to health and other services [69]. COVID-19 is also providing challenges for disease surveillance and control programs as well as public health systems in Colombia and this is likely to have long lasting impact on vector-borne diseases [70,71]. It is therefore more imperative than ever that we more fully understand the dynamics of these important diseases which are likely to escalate over the coming years.

The arrival of chikungunya and Zika in 2015 established a co-transmission of three different arboviruses by *Ae*. *aegypti*. Unexpectedly, a reduction in dengue cases was found in parallel to the spike in Zika cases in the year of the Zika epidemic of 2016 in Cúcuta. The same location in the years prior to the Zika outbreak had consistently presented high incidences of dengue. Moreover, in Cúcuta the incidence of both dengue and severe cases of dengue were lower in 2017, the year following the Zika epidemic, than in any of the 10 years prior. The decline of dengue following Zika reported in this study agrees with the overall decline in dengue incidence across the whole of Colombia [72,73] and has also been observed in other dengue endemic countries across the Americas [73,74]. In 2017 the total number of dengue cases across the Americas was lower than any of the 10 previous years [75] with a 73% decline between 2016 and 2017 alone [74].

Changes in epidemiological surveillance systems can cause the identification of inaccurate patterns of disease incidence. However, we did not observe any indication of significant changes to the surveillance system used to report *Aedes* borne viruses to SIVIGILIA, the database used here. However, the circulation of multiple viruses in the same localities at the same time does provide challenges for surveillance systems. Clinical presentation of Zika is very similar to that of dengue [27,76] and this can cause cases to be misidentified when laboratory testing is not conducted. We note that although the cases analysed in this study are all confirmed cases, confirmation is not always done by laboratory testing but also by epidemiological links. Misidentification could therefore explain the increase in dengue that was observed in Bello and Moniquirá in 2016, where low incidence of Zika was reported. Increased dengue incidence in 2015 and 2016 in other regions has also been reported and attributed to potential misidentification of Zika [74]. Misidentification could also be a factor in the observed decline of dengue in Cúcuta in 2016, with dengue cases being misidentified as Zika during the Zika outbreak.

Coinfection of the primary vector *Ae*. *aegypti* with multiple arboviruses (i.e. DENV, CHIKV, ZIKV) has been reported following laboratory exposure [77–82], with an enhanced susceptibility to ZIKV (PMID 33214283). *Aedes* mosquitoes have also been shown capable of transmitting more than one arbovirus in a single biting event [78,82,83]. Although coinfection has yet to be found in wild *Ae*. *aegypti* [83]. Coinfection of multiple *Aedes* borne viruses has been reported in mammalian hosts including humans [84–90]. In Colombia patients have been diagnosed with DENV-CHIKV [66,91,92], DENV-ZIKV [66,92], CHIKV-ZIKV [66,92] and DENV-CHIKV-ZIKV [27,67] coinfections. However, the frequency of DENV-ZIKV co-infections seems low at 8.8% [92]. DENV and ZIKV co-transmission in mice through the bite of *Ae*. *aegypti* mosquitoes showed preferential transmission of ZIKV [82].

Host cross-immunity of ZIKV and DENV could have been a contributing factor in the dengue declines observed in this study. The observed decline in dengue cases following Zika outbreaks reported within this study and in others across the Americas suggest that there may be cross-immunity between ZIKV and DENV in humans [72–74, 93–119]. Flavivirus immunity involves a T cell response and studies have reported cross-reactivity of CD4+ and CD8+ T cells to both DENV and ZIKV [120–124]. Cross-reactivity of antibodies and T cells and cross-immunity from Zika, although not necessarily conferring cross-protection, has been presented as the most probable reason explaining the decline of dengue across the Americas in 2017 [74].

Specific climatic factors associated here significantly affected disease incidence. We found significant co-relationship between average temperature and wind speed with disease transmission, with a peak at around 2m/s, consistent with findings of previous studies [125–128]. *Ae*. *aegypti* has a small flight range of 200 m and high wind speeds reduce mosquito flight distances while low winds mean reduced dispersion of mosquitoes. We found the optimum wind speed to be around 2 m/s which is in line with current knowledge of mosquito flight [125,129,130]. A significant relationship was also found between increasing minimum temperature and decreasing disease incidence, contrasting to the findings of some other studies [130–133]. Exposure of *Ae*. *aegypti* to fluctuations at low temperatures has been associated with shorter DENV extrinsic incubation periods (EIP) and increased virus dissemination from the midgut when compared to exposure to constant temperatures with the same mean [134].Reduced EIP and increased virus dissemination increase transmission potential [23]and could explain the relationship between increasing minimum temperature and decreasing disease incidence observed in this study. Climate variables can be used to build predictive models to anticipate when outbreaks of dengue, chikungunya and Zika are likely to occur [135–138]. This is useful in the prioritisation of vector-control resources. Recent modelling studies have reported an increase in the incidence and geographical spread *Ae*. *aegypti* borne viruses when using climate change simulation models [139–143]. This highlights the importance of consideration of environmental factors when assessing risk of vector-borne disease [144].

We report differences in measurements of socio-economic variables between Bello, Cúcuta and Moniquirá. Bello, the municipality with the lowest burden of *Aedes* borne viruses also had the lowest poverty index, whilst Cúcuta and Moniquirá were much higher in both disease incidence and multidimensional poverty. Higher incidence of *Aedes* borne disease has been associated with lower socio-economic status and higher poverty levels [145–152]. Cúcuta had the highest rate of critical overcrowding. Overcrowding has been reported to be an important contributing factor to dengue incidence [144–146,153]. Inadequate sanitation, and no access to improved sources of water are both well-known contributing factors in increasing burden of *Aedes* borne disease due to the ecology of *Aedes* mosquitoes [33,144,148–150,154]. These socio-economic risk factors were highest in Moniquirá where there were also high levels of low educational achievement and illiteracy. Illiteracy and low educational level have previously been associated with increased vulnerability to dengue in Colombia and Brazil [155,156].

This study has potential limitations, due to the nature and availability of the data used. Co-infections were unable to be analysed due to the inability to identify these within the data set, despite co-infections likely occurring during our study period. The investigation into the effects of socioeconomics on disease incidence are limited by the unavailability of temporal data, therefore changes in socioeconomics and their impact on disease burden over time could not be analysed.

Having different ecosystems Bello, Cúcuta and Moniquirá presented a valuable opportunity to explore longitudinal arboviral disease incidence over 11 years that encompassed a Zika epidemic. Chikungunya was the only disease for which incidence did not significantly differ between the three municipalities. Cúcuta had the greatest disease incidence having the most favourable climatic factors and greater poverty index but as it borders with Venezuela mass movement of people is also suggested to be a contributing factor. Climatic factors associated with disease incidence were precipitation, average humidity, temperature and wind speed. Co-transmission of dengue and Zika during the epidemic led to a significant reduction of dengue cases in Cúcuta where dengue had previously been high. This significant finding warrants further investigation. Where the poverty index was low, as in Bello, so was the disease incidence. Socio-economic factors such as barriers to health and childhood services, inadequate sanitation, poor housing and poor water supply were implicated as drivers of disease transmission. *Aedes aegypti* and *Ae*. *albopictus* are increasing their geographical range and climate change is predicted to alter the distribution of these vectors and hence disease risk. Arboviral epidemiology is further complicated by humanitarian crises (e.g. political and economic crises Venezuela leading to mass migration) and the COVID-19 pandemic which reinforces the urgency for understanding the dynamics of these global health problems. Context dependent and actionable understanding of the drivers for disease transmission that consider local dynamics, both climatic and socio-economic, should contribute to the design of more effective vector mosquito control programmes [157].

## Supporting information

S1 Table Disease, climate and socio-economic variables used in the analysis. (XLSX)

S2 Table Indicators used for the calculation of MPI in Colombia including their respective weighting. Calculations are completed at household level.
(XLSX)

S3 Table Disease incidence data at department level. Yearly incidence of dengue, severe dengue, chikungunya and Zika per 100,000 people in each Colombian department from 2007–2017.
(XLSX)

S4 Table ANOVA and Post-Hoc Tukey Analysis of Climate Data. The results of the anova and post-hoc Tukey analysis conducted on the climate parameteres to test for differences between municipalites.
(XLSX)
